# A murine model for human early/immature T-cell precursor acute lymphoblastic leukemia (EITP ALL)

**DOI:** 10.18632/oncoscience.567

**Published:** 2022-10-24

**Authors:** Vijay Negi, Peter D. Aplan

**Affiliations:** ^1^Genetics Branch, Center for Cancer Research, National Cancer Institute, National Institutes of Health, Bethesda, MD 20892, USA

**Keywords:** early thymocyte precursor (ETP), leukemia, IDH2, NUP98, NHD13

## Abstract

Early/immature T cell precursor acute lymphoblastic leukemia (EITP ALL) represents a subset of human leukemias distinct from other T-ALL, and associated with poor prognosis. Clinical studies have identified chromosomal translocations involving the *NUP98* gene and point mutations of *IDH* genes as recurrent mutations in patients with EITP-ALL. In a recent study using genetically engineered mice, we demonstrated that cooperation of an *Idh2^R140Q^* mutation with a *NUP98-HOXD13* (*NHD13*) fusion gene resulted in EITP-ALL. Highlights of this double transgenic mouse model included the similarity of the immunophenotypic, mutational and gene expression landscape with human EITP-ALL. Additional studies showed that the* Idh2^R140Q^*/*NHD13 EITP-ALL* are sensitive to selective mutant *IDH2* inhibitors *in vitro*, leading to the possibility that these mice can serve as a useful model for the study of EITP ALL development and therapy.

Early T-cell precursor (ETP) leukemia and early immature T-ALLs represent a group of human leukemia that have an immunophenotype and gene expression profile that is distinct from other T-ALLs, and has historically been associated with poor prognosis [1, 2]. Because ETP leukemias and immature T-ALLs have similar immunophenotype and gene expression profile we and others elected to group them as early/immature T cell precursor (EITP) ALL [2, 3]. The characteristic immunophenotype is negative for markers of mature thymocytes (CD4 and CD8) but positive for markers of myeloid or hematopoietic stem cells.

Highly specific gain of function mutations in isocitrate dehydrogenase 1 or 2 (*IDH1/2*) were identified in patients with acute myeloid leukemia (AML) over a decade ago [4, 5], and have more recently been associated with T-cell leukemias [2, 3, 6, 7], especially EITP [8]. In efforts to develop *in vivo* models for IDH1/2 leukemia, several investigators have developed genetically engineered mice that express mutant forms of *Idh1* or *Idh2* [3, 9, 10]. In general, these mice did not develop leukemia, suggesting that additional, complementary mutations may be required for oncogenic transformation initiated by an *Idh1/2* mutation.

Coincidentally, whole exome sequencing of AML which developed in mice that expressed a *NUP98-HOXD13 (NHD13)* transgene revealed that 21% of these samples had acquired an Idh1p.R132H mutation, suggesting that an *NHD13* transgene might collaborate with an Idh1/2 mutation to generate AML [11]. Since IDH2p.R140Q is the most common IDH mutation seen in patients with AML [4], we predicted that the combination of these two mutations would lead to AML, and generated *Idh2^R140Q^* transgenic mice that were crossed with *NHD13* transgenic mice to test this hypothesis. As anticipated, we found that the onset of leukemia was significantly accelerated in *Idh2^R140Q^/NHD13* double transgenic mice compared to single transgenic *NHD13* or *Idh2^R140Q^* mice [3]. Surprisingly, the vast majority of leukemias that developed in *Idh2^R140Q^/NHD13* double transgenic mice were not AML, but rather an immature T-cell leukemia that displayed an immunophenotype which was consistent with either DN1 thymocytes (CD4^−^CD8^−^CD44^+^CD25^−^CD90^−^Kit^+^), DN2 thymocytes, (CD4^−^CD8^−^CD44^+^CD25^+^CD90^+^Kit^−^), or an immunophenotype intermediate between DN1 and DN2. Additionally, lack of surface CD3 (but presence of cytoplasmic CD3), and the presence of clonal *Tcrb* DJ (but not complete VDJ) gene rearrangements suggested that the murine *Idh2^R140Q^/NHD13* DN1/DN2 leukemias were similar to human EITP-ALL.

Early immature thymic progenitors that retain both lymphoid and myeloid lineage potential have been identified as the target cell population for EITP-ALL transformation [1, 12]. Examination of young, clinically healthy (i.e, no evidence of leukemia) *Idh2^R140Q^/NHD13* double transgenic mice showed a severe block in thymocyte maturation, and an expansion of DN thymocytes with oligoclonal *Tcrb* DJ rearrangement. Although *NHD13* transgene alone impairs thymocyte differentiation [3, 13], this is potentiated by the addition of the *Idh2^R140Q^* transgene, resulting in a severe differentiation block at the DN2 to DN3 transition. The differentiation block characterized at an immunophenotype level was also evident at a transcriptional level as *Idh2^R140Q^/NHD13* DN1/DN2 leukemias were enriched for genes expressed in DN1 thymocytes as compared to DN3 thymocytes. Taken together, these findings support the hypothesis that *Idh2^R140Q^/NHD13* leukemias originated from early T cell precursors in the thymus, similar to human EITP-ALL [1].

The genomic landscape of human EITP-ALL has been recently characterized [8, 12], and mutations that are more prevalent in EITP-ALL as compared to non-EITP T-ALL have been identified. Using whole exome sequencing we were able to show that acquired mutations in human EITP-ALL (such as *KRAS, NRAS, PTPN11, JAK3, SH2B3, SETD2,* and *EZH2*) were enriched in *Idh2^R140Q^/NHD13* DN1/DN2 T-ALL, and *NOTCH1* mutations, which are less common in EITP T-ALL were also less common in the *Idh2^R140Q^/NHD13* DN1/DN2 T-ALL. Finally, gene set enrichment analysis (GSEA) showed that the gene expression profile of *Idh2^R140Q^/NHD13* DN1/DN2 T-ALL was similar to that of human EITP-ALL, further reinforcing the potential of this mouse model in elucidating transformation pathways relevant for understanding human EITP-ALL (Figure 1).

**Figure 1:**
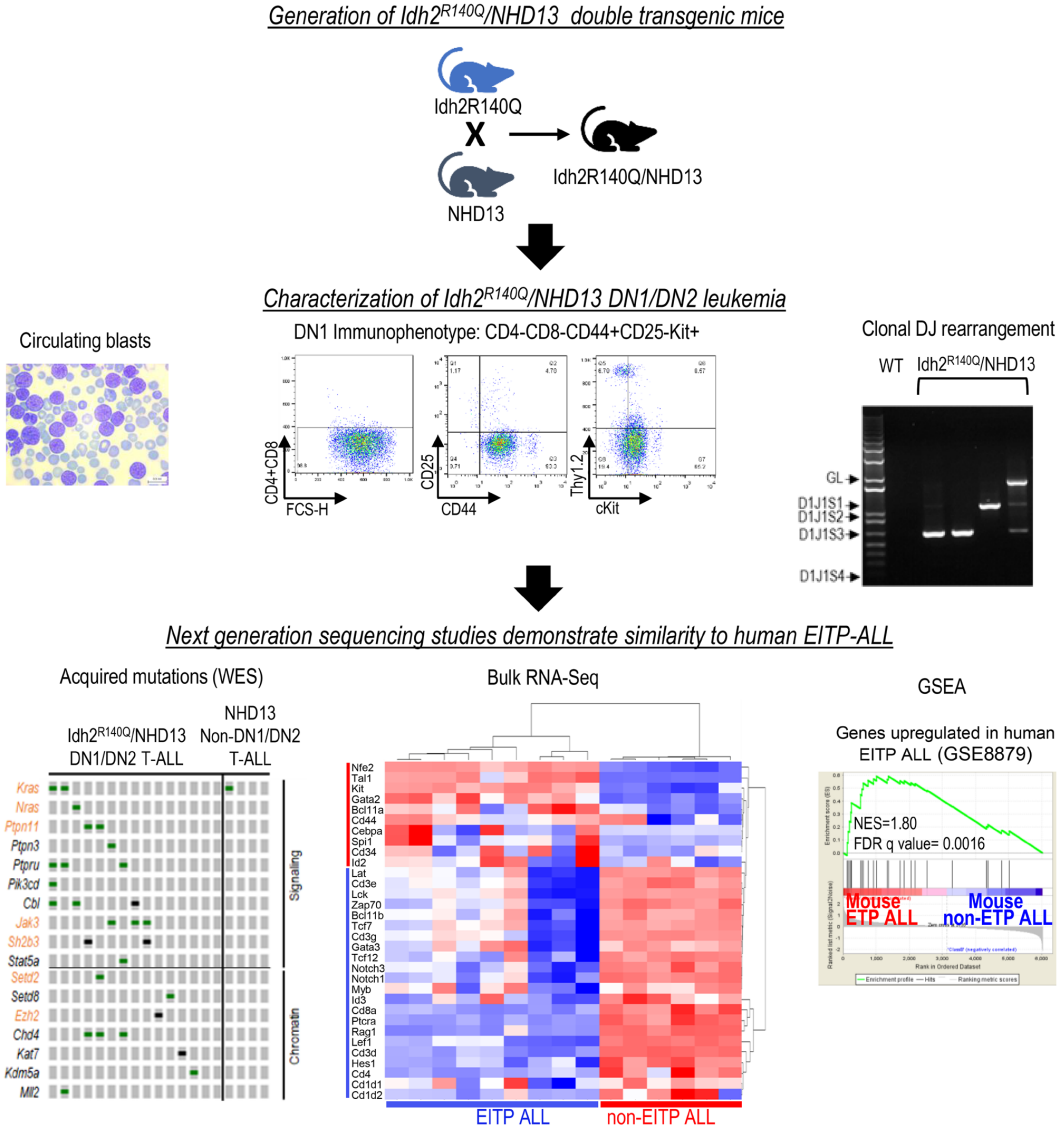
*Idh2^R140Q^*/*NHD13* double transgenic mice develop EITP ALL resembling the human disease. Top, Generation of *Idh2^R140Q^/NHD13* mice. Middle, Leukemic cells display blast morphology, DN1 immunophenotype, and clonal *Tcrb* DJ rearrangement. GL, germline (non-rearranged) *Tcrb.* Bottom, Acquired mutations identified by whole exome sequencing (WES) in *Idh2^R140Q^/NHD13* leukemia compared to those in *NHD13*-only leukemia. Mutations in orange are common in human EITP. RNA-seq from murine EITP ALL (*Idh2^R140Q^/NHD13*) compared to murine non-EITP ALL. GSEA shows similarity between genes enriched in human and mouse EITP.

Enasidenib (AG-221) is a potent selective inhibitor of the mutant IDH2 enzyme which has recently been approved for treatment of relapsed or refractory AML patients with *IDH2* mutations [14]. Using an OP9-DL1 co-culture system we established an immortalized *Idh2^R140Q^/NHD13* DN cell line and found that treatment of these immortalized cells with AG-221 led to marked decrease in cell proliferation suggesting targeting *IDH2* mutations may be an effective treatment for EITP-ALL as well as AML.

The study by Goldberg and colleagues [3] demonstrated that collaboration of an *IDH2* mutation with a *NUP98-HOXD13* translocation leads to a highly penetrant EITP-ALL by targeting early thymic progenitor cells. In the context of human disease, it is important to note that both *NUP98* translocations and *IDH1/2* mutation have been reported as recurrent events in EITP-ALL [6–8, 15, 16]. Additionally, the report [3] demonstrated that the *Idh2^R140Q^/NHD13* DN1/DN2 T-ALL recapitulates human EITP-ALL in terms of immunophenotype, *Tcrb* gene rearrangements, gene expression profile, and landscape of acquired mutations. We predict that the *Idh2^R140Q^/NHD13* mouse model will serve as an excellent tool to study EITP biology and identify therapies for patients with EITP leukemia.

## References

[1] Coustan-Smith E, Mullighan CG, Onciu M, Behm FG, Raimondi SC, Pei D, Cheng C, Su X, Rubnitz JE, Basso G, Biondi A, Pui CH, Downing JR, Campana D. Early T-cell precursor leukaemia: a subtype of very high-risk acute lymphoblastic leukaemia. Lancet Oncol. 2009; 10:147–56. 10.1016/S1470-2045(08)70314-0. 19147408 PMC2840241

[2] Haydu JE, Ferrando AA. Early T-cell precursor acute lymphoblastic leukaemia. Curr Opin Hematol. 2013; 20:369–73. 10.1097/MOH.0b013e3283623c61. 23695450 PMC3886681

[3] Goldberg L, Negi V, Chung YJ, Onozawa M, Zhu YJ, Walker RL, Pierce R, Patel DP, Krausz KW, Gonzalez FJ, Goodell MA, Rodriguez BAT, Meltzer PS, Aplan PD. Mutant *Idh2* Cooperates with a *NUP98-HOXD13* Fusion to Induce Early Immature Thymocyte Precursor ALL. Cancer Res. 2021; 81:5033–46. 10.1158/0008-5472.CAN-21-1027. 34321240 PMC8487989

[4] Marcucci G, Maharry K, Wu YZ, Radmacher MD, Mrózek K, Margeson D, Holland KB, Whitman SP, Becker H, Schwind S, Metzeler KH, Powell BL, Carter TH, et al. IDH1 and IDH2 gene mutations identify novel molecular subsets within de novo cytogenetically normal acute myeloid leukemia: a Cancer and Leukemia Group B study. J Clin Oncol. 2010; 28:2348–55. 10.1200/JCO.2009.27.3730. 20368543 PMC2881719

[5] Paschka P, Schlenk RF, Gaidzik VI, Habdank M, Krönke J, Bullinger L, Späth D, Kayser S, Zucknick M, Götze K, Horst HA, Germing U, Döhner H, Döhner K. IDH1 and IDH2 mutations are frequent genetic alterations in acute myeloid leukemia and confer adverse prognosis in cytogenetically normal acute myeloid leukemia with NPM1 mutation without FLT3 internal tandem duplication. J Clin Oncol. 2010; 28:3636–43. 10.1200/JCO.2010.28.3762. 20567020

[6] Andersson AK, Miller DW, Lynch JA, Lemoff AS, Cai Z, Pounds SB, Radtke I, Yan B, Schuetz JD, Rubnitz JE, Ribeiro RC, Raimondi SC, Zhang J, et al. IDH1 and IDH2 mutations in pediatric acute leukemia. Leukemia. 2011; 25:1570–77. 10.1038/leu.2011.133. 21647154 PMC3883450

[7] Van Vlierberghe P, Ambesi-Impiombato A, Perez-Garcia A, Haydu JE, Rigo I, Hadler M, Tosello V, Della Gatta G, Paietta E, Racevskis J, Wiernik PH, Luger SM, Rowe JM, et al. ETV6 mutations in early immature human T cell leukemias. J Exp Med. 2011; 208:2571–79. 10.1084/jem.20112239. 22162831 PMC3244026

[8] Simonin M, Schmidt A, Bontoux C, Dourthe MÉ, Lengliné E, Andrieu GP, Lhermitte L, Graux C, Grardel N, Cayuela JM, Huguet F, Arnoux I, Ducassou S, et al. Oncogenetic landscape and clinical impact of IDH1 and IDH2 mutations in T-ALL. J Hematol Oncol. 2021; 14:74. 10.1186/s13045-021-01068-4. 33941203 PMC8091755

[9] Ogawara Y, Katsumoto T, Aikawa Y, Shima Y, Kagiyama Y, Soga T, Matsunaga H, Seki T, Araki K, Kitabayashi I. IDH2 and NPM1 Mutations Cooperate to Activate Hoxa9/Meis1 and Hypoxia Pathways in Acute Myeloid Leukemia. Cancer Res. 2015; 75:2005–16. 10.1158/0008-5472.CAN-14-2200. 25795706

[10] Sasaki M, Knobbe CB, Munger JC, Lind EF, Brenner D, Brüstle A, Harris IS, Holmes R, Wakeham A, Haight J, You-Ten A, Li WY, Schalm S, et al. IDH1(R132H) mutation increases murine haematopoietic progenitors and alters epigenetics. Nature. 2012; 488:656–59. 10.1038/nature11323. 22763442 PMC4005896

[11] Goldberg L, Gough SM, Lee F, Dang C, Walker RL, Zhu YJ, Bilke S, Pineda M, Onozawa M, Jo Chung Y, Meltzer PS, Aplan PD. Somatic mutations in murine models of leukemia and lymphoma: Disease specificity and clinical relevance. Genes Chromosomes Cancer. 2017; 56:472–83. 10.1002/gcc.22451. 28196408 PMC5399546

[12] Zhang J, Ding L, Holmfeldt L, Wu G, Heatley SL, Payne-Turner D, Easton J, Chen X, Wang J, Rusch M, Lu C, Chen SC, Wei L, et al. The genetic basis of early T-cell precursor acute lymphoblastic leukaemia. Nature. 2012; 481:157–63. 10.1038/nature10725. 22237106 PMC3267575

[13] Choi CW, Chung YJ, Slape C, Aplan PD. A NUP98-HOXD13 fusion gene impairs differentiation of B and T lymphocytes and leads to expansion of thymocytes with partial TCRB gene rearrangement. J Immunol. 2009; 183:6227–35. 10.4049/jimmunol.0901121. 19841179 PMC3422874

[14] Yen K, Travins J, Wang F, David MD, Artin E, Straley K, Padyana A, Gross S, DeLaBarre B, Tobin E, Chen Y, Nagaraja R, Choe S, et al. AG-221, a First-in-Class Therapy Targeting Acute Myeloid Leukemia Harboring Oncogenic *IDH2* Mutations. Cancer Discov. 2017; 7:478–93. 10.1158/2159-8290.CD-16-1034. 28193778

[15] Van Vlierberghe P, Ambesi-Impiombato A, De Keersmaecker K, Hadler M, Paietta E, Tallman MS, Rowe JM, Forne C, Rue M, Ferrando AA. Prognostic relevance of integrated genetic profiling in adult T-cell acute lymphoblastic leukemia. Blood. 2013; 122:74–82. 10.1182/blood-2013-03-491092. 23687089 PMC3701905

[16] Liu Y, Easton J, Shao Y, Maciaszek J, Wang Z, Wilkinson MR, McCastlain K, Edmonson M, Pounds SB, Shi L, Zhou X, Ma X, Sioson E, et al. The genomic landscape of pediatric and young adult T-lineage acute lymphoblastic leukemia. Nat Genet. 2017; 49:1211–18. 10.1038/ng.3909. 28671688 PMC5535770

